# An Overview of the Available Intervention Strategies for Postural Balance Control in Individuals with Autism Spectrum Disorder

**DOI:** 10.1155/2022/3639352

**Published:** 2022-11-21

**Authors:** Rabeeh Hariri, Amin Nakhostin-Ansari, Fatemeh Mohammadi, Amir Hossein Memari, Iman Menbari Oskouie, Afarin Haghparast

**Affiliations:** Sports Medicine Research Center, Tehran University of Medical Sciences, Tehran, Iran

## Abstract

**Background:**

Postural instability is a prevalent issue among individuals with autism spectrum disorder (ASD) that affects the development of their perceptual-motor skills and social functioning. Visual and somatosensory processing deficits, hypotonia, basal ganglia dysfunction, and anxiety are some of the concurrent disorders in individuals with ASD. Nevertheless, a definite management protocol for postural instability in ASD has not been introduced yet. Hence, we aim to shed light on the available intervention strategies for postural instability in individuals with ASD.

**Methods:**

Even though several studies have been conducted on the effects of various interventions for balance control in individuals with ASD, no study has compared their efficacy, limitations, and clinical implications.

**Results:**

This review discusses diverse proposed interventions contributing to ASD postural instability, including martial arts, water-based interventions, animal-assisted therapies, trampoline, balance training, vestibular therapy, transcranial direct current stimulation, sports, play, and active recreation for kids (SPARK), and square-stepping exercise (SSE).

**Conclusion:**

Enhancing motor skills, cerebellum function, and sensory input integration were some of the main mechanisms of these interventions to improve balance control in ASD. Some interventions, such as water-based exercises and video games, were enjoyable for children with ASD and could raise their treatment adherence. In most studies, small sample sizes and the lack of a control group represented their major limitations. Therefore, future well-designed randomized controlled trials are required to assess the effects of available interventions on postural control in ASD.

## 1. Introduction

Autism spectrum disorders (ASD) comprise complex neurodevelopmental conditions characterized by communication deficits, reciprocal social interactions, restricted interests, and repetitive behavior patterns [1]. Although motor deficits have been identified as a limiting factor in daily motor skills, they have not yet been included in the diagnostic features of ASD in the DSM-5 criteria [2]. The Centers for Disease Control and Prevention (CDC) has published that the estimated prevalence of ASD in the US population was about 1 : 54 children aged eight years (about 1.85% of children) [3, 4]. Moreover, the World Health Organization (WHO) 2010 documented its worldwide prevalence of about 0.76% (among nearly 1.16% of the entire child population) [5]. However, the prevalence of motor deficit in individuals with ASD has been estimated near 24–79% regarding their age, highlighting its clinical value for further research [6]. A study in South Korea has demonstrated that ASD carries a high financial cost ($2,700,596 in 2008 and $9,645,503 in 2015, with 72.3% and 27.7% attributing to direct and indirect costs, respectively). Compared to the prevalence mentioned, its economic burden has considerably increased over time [7]. In light of this, many interventions have been introduced to reduce the burden of ASD. Among them, some are recognized for improving their poor motor performance. However, no subsequent update has been established for all available management strategies. There is a lack of consensus on the treatment of choice for those with postural instability. Therefore, we aim to shed light on the interventions previously described as treatment options for the postural balance of individuals with ASD to increase awareness of managing motor involvement in ASD.

## 2. Balance Disorder in Individuals with ASD

ASD predisposes individuals to developmental impairment, gastrointestinal problems, and sensory abnormalities [8]. However, motor disturbances should also raise suspicion toward ASD diagnosis [2]. The impaired motor function is correlated with a high level of repetitive behavior patterns and a low level of expressive language [9].

Motor disruptions can range from dynamic to static postural challenges [2]. Moreover, decreased proficiency in targeted strength, speed, agility, and coordination may also serve as a hint for identifying ASD [10]. Stance tasks using force plates or Wii balance boards are one of the methods to assess postural control [11, 12]. Swaying at higher velocity during standing position and increased mediolateral sway during walking tests are the clinical features of postural instability [11]. A recent review has revealed that postural control difficulties in ASD can arise from visual and somatosensory processing deficits [13, 14]. Hypotonia may also result in poor balance control in children with ASD. It can exhibit single-leg stance test impairment associated with a deficit in basal ganglia function [15, 16]. Furthermore, anxiety can lead to activity avoidance, altered primary sensory processing, and impaired balance control secondary to sensory inputs [17]. It has been reported that balance performance in those with ASD depends on the severity of the symptom and body mass index (BMI) [18–20]. However, symptom severity modifies the association between balance and BMI [20].

Previous literature has investigated that impaired motor functions in childhood can constrain social interaction due to decreased social participation [12]. As a result, the postural control deficit can substantially impact the development of perceptual-motor skills and social functioning in ASD [21, 22]. Therefore, a management guideline for balance control is required for individuals with ASD. Although many strategies have been proposed to treat the underlying causes of motor disturbances, the potential benefits of each intervention are still far from being understood. [9, 12].

## 3. Intervention to Improve Balance

### 3.1. Martial Art

Different forms of martial arts have been frequently used to improve postural balance in individuals with ASD. These art styles are usually welcomed by the youth population, with the development of body awareness being the most common mechanism of action [23, 24].

Kata, a karate division consisting of movements in a specific order, can improve static and dynamic balance in individuals with ASD. Performing rapid changes in direction and technical movements in kata are challenges for postural balance. So, practicing such exercises may enhance the patient's balance, especially static stability, in the long term [25]. Kata training may also impact balance control by improving body equilibrium, increasing muscle strength, correcting body alignment, and enhancing body awareness [23]. However, these satisfactory results have been achieved among 8–14-year-old boys [23]. Ansari et al. have shown that in those with ASD, ten weeks of kata training in 8–14-years-old boys with ASD who were at level 1 or 2 of severity according to Gars-2 can affect their performance in static and dynamic balance through the walking heel-to-toe and stork tests, respectively [23].

Tai Chi Chuan training has also been reported to enhance balance in individuals with ASD [24]. Improved balance performance can be achieved through Tai Chi Chuan training through cognitive-motor exercise, neuromuscular coordination enhancement, and anxiety control, which is highly prevalent among ASD cases [26–28]. Besides, Tai Chi Chuan training could also affect the acquisition of motor skills, somatosensory systems, and adaptive systems, which all play a role in the balance progression in ASD [24].

Sarabzadeh et al. have found that six weeks of Tai Chi Chuan training, three sessions per week, can improve ball skills and static and dynamic balance in 6–12-years-old children with ASD using the second edition of the movement assessment battery for children [24].

As another form of martial arts, Taekwondo has been evaluated to determine whether it can improve balance in individuals with ASD. Kim et al. have revealed that 16 sessions of Taekwondo practice in 8–14-years-old children with ASD can improve their performance in the right single-leg stance test compared to controls with closed eyes. However, there were no other statistically significant differences between groups in their performance in single-leg stance tests with eyes open or closed. Also, performances in the double-leg stance test on stable and unstable surfaces with open or closed eyes and functional balance did not significantly change between groups [29]. The outcomes of this study should be carefully interpreted due to the small sample size and lack of statistically significant results, and future studies with larger sample sizes are suggested. An increase in knee extensor muscle strength and standing on one leg during kicking can be considered a reason for improving the single-leg stance test after practicing Taekwondo [29, 30].

Regarding the discussed articles, martial arts practice can effectively improve static and dynamic balance in ASD cases. Moreover, children have demonstrated a higher willingness to participate in such programs [29], resulting in increased adherence to the treatment. However, all the studies mentioned have been limited to youth participants [23, 24, 29]. ASD is a chronic condition [31], and both younger and older individuals with ASD may suffer from postural balance impairment, considering the underdevelopment of postural control in these individuals [32]. Therefore, further research is required to determine whether martial arts are effective in improving postural balance in older subjects. Moreover, according to their small sample sizes [23, 24, 29], studies with larger sample sizes in young individuals with ASD are recommended.

### 3.2. Water-Based Interventions

Water-based exercises differ from land-based ones, considering the water's frictional force and buoyancy. Consequently, the subjects move in the water with more difficulty than on land, improving their muscles' strength, which helps to enhance balance [33, 34]. Therefore, some interventions have benefited from the characteristics of water and water-based exercises to improve postural balance in individuals with ASD [23, 35]. In addition to the mechanical characteristics of water, training in such environments may be more pleasant for children with ASD, promoting their adherence to the treatment [36]. Yilmaz et al. demonstrated that ten weeks of swimming could enhance the motor performance of a patient with ASD in a single-leg stance test with eyes open or closed. However, this study was a case report, so more research is needed [35]. Moreover, Ansari et al. found that ten weeks of water exercise can improve static and dynamic balance in the male population with ASD by facilitating postural control, strengthening muscles, and adapting the CNS [23, 35–38]. Regarding its findings, aquatic exercises are considered inexpensive and enjoyable interventions for postural balance enhancement in those with ASD. The mentioned study was limited to young individuals. Thus, further research is needed to comprehensively evaluate aquatic training in balance control for both sexes and older age-ranged ASD cases. Special attention must be paid to the low percentage of physical therapists tempted to use aquatic exercises to rehabilitate individuals with ASD [39].

### 3.3. Animal-Assisted Therapies

Animal-assisted therapies have been utilized for occupational therapy in different neurological conditions, and they have recently gained popularity in occupational therapy of individuals with ASD [40–45]. These interventions positively influence their subjects' willingness to engage in routine life tasks [46]. Animal-assisted therapies positively affect motor skills, sensory system function, and communication skills in those with ASD and have promising effects on their postural balance [42–45, 47].

In Thailand, elephant-assisted therapy has been reported to improve a wide range of ASD complications, including postural balance [42, 43]. Satiansukpong et al. designed an intervention using elephant-assisted therapy. They enrolled four boys with ASD who were 11–19 years old, and one elephant was assigned to each. Their intervention contained different tasks, such as becoming oriented about elephants, taking care of elephants, riding elephants, and relaxing. After the intervention, postural control and dynamic and static balance were improved in all four individuals [43]. Sensory integration and sensory processing enhanced after the intervention. Furthermore, the vestibular system may be stimulated by tactile sensation during riding [43]. Advances in the sensory system's function may lead to better postural control in those with ASD [48, 49]. Elephant-assisted therapy may also improve postural balance by enhancing cerebellum function [50, 51].

Elephant-assisted therapy also positively affects adaptive behavior, communication skills, and socialization in individuals with ASD. However, limited access to elephants across countries and regions and their high price constrain its feasibility. Therefore, countries may benefit from similar culturally adapted animal-assisted interventions, which may share principles with elephant-assisted therapy.

Nuntanee et al. designed a motorized elephant-assisted therapy to improve its feasibility by using artificial elephants in their study. Their interventions included washing the elephant, riding it, and playing games while sitting on the elephant. Also, they asked the subjects to get on and off the artificial elephants. They investigated and found that postural sway was decreased in all directions in the intervention group during their stance on foam or the floor with eyes open or closed. However, changes were not statistically significant in a few circumstances due to the small sample size [42]. Decreased postural sway has indicated an improvement in balance control after the intervention through a similar mechanism to elephant-assisted therapy [42, 52]. Moreover, motorized elephant-assisted therapy might be more accessible and feasible because there is no need for an entire day to perform all tasks, unlike elephant-assisted therapy.

Ajzenman et al. designed a hippotherapy program with progressive difficulty, including five components: motor control, social and communication skills, cognition, and interactive games. After 12 treatment sessions, they demonstrated significantly improved balance control and routine life activity engagement in 5–12-years-old children with ASD [45]. Riding horses could impose challenges on the subjects with every step that horses took, especially when riding in different directions, at different speeds, and during episodes of stopping and starting riding. These challenges may all lead to an enhancement in trunk stability, cerebellum, and equilibrium function, leading to improved postural control [45, 53, 54]. Achieving new skills and improving postural balance may also have practical implications on subjects' lives, as they may motivate them to engage in daily activities more and consequently be more socialized [45]. Since the mentioned study was a pilot, further studies are needed to evaluate and determine hippotherapy's effects on individuals with ASD. However, the various positive effects seen in Ajzenman et al.'s study suggest hippotherapy as a potentially effective candidate in occupational therapy programs for individuals with ASD.

Wuang et al. designed a simulated developmental horse-riding program to enhance sensory integration and motor proficiency in 6–10-years-old children with ASD [44]. The program was individualized based on the subjects' interests and abilities. Besides, a progressive nature was found in the program because the subjects were instructed to perform more complex tasks after reaching each milestone. The program included simple exercise and limb movements, riding Joba, a horse-riding simulator, in different positions, and playing a game on Joba. The subjects also received their routine occupational therapy during the intervention. After 40 sessions of treatment, the postural balance was significantly improved in those with ASD [44]. They have also reported that sensory integration, gross motor, and fine motor skills were enhanced, which may also be associated with their balance control [49, 55, 56].

### 3.4. Trampoline

Lourenco et al. conducted a study to evaluate whether trampoline exercise can influence balance control in ASD. They added further coordinated movements and cognitive tasks to simply jumping on a trampoline to make it a more challenging exercise. After 20 training sessions, the study revealed a significant improvement in the tandem walk test, indicating better dynamic balance in the intervention group compared to controls. The study's results demonstrated that trampoline exercise could improve balance in children 4–11 years old with mild to moderate ASD [57]. Practicing motor coordination can improve performance on the tandem gait test in individuals with ASD, in which their vestibular function is assessed [57–59]. The study had an insufficient sample size and used only a single balance test, which evaluated dynamic balance. So, supplementary studies with larger sample sizes and more comprehensive tests are needed to evaluate the effect of trampoline exercise on balance control in ASD.

### 3.5. Balance Training

Balance training may have several benefits for individuals with ASD, as it can affect cerebellum function, motor skills, and muscle strength, enhancing postural balance [60, 61]. Cheldavi et al. designed a progressive balance training program and evaluated its effects on postural sway in 7–10-years-old boys with ASD [60]. Their program included four types of training, such as standing on one leg (with eyes open or closed, on foam or hard surface), walking in a determined path (in a linear or curved path, heel-toe or gait), maintaining balance on one leg while flexing, extending, abducting, or adducting the other leg (with eyes open or closed, on foam or hard surface), and standing on a balance board (with eyes open or closed, with or without help). The authors arranged a progressive complexity of each type of training; therefore, the subjects could perform more complicated tasks based on their desire and ability. After six weeks of training (three sessions per week), subjects who had participated in the training program had less sway in the mediolateral and anteroposterior directions. They also had a lower overall velocity of the center of pressure, indicating more stability and better postural balance [60].

Caldani et al. conducted a study to evaluate whether short-training rehabilitation programs affect postural control parameters. In this regard, they prepared two postural control training exercises, including the buoy and the crowd. The results have revealed an improvement in the postural balance of individuals with ASD (mean age of 11.7 years) after a short postural rehabilitation training program. However, further studies are requested due to their small sample size [62].

Travers et al. utilized video games to design a program enhancing the postural balance in ASD, considering the popularity of these video games among ASD cases [63, 64]. Their program was individualized based on the balance ability of each participant. It consisted of two sets of training video games, with Wii fitness games between the two sets. In balance training sessions, the subjects were asked to perform three to six sets of ninja poses and maintain the correct position for up to twice the defined goal of 5–120 seconds. Red dots on the screen indicated incorrect positions, and the subjects had to correct their positions until the dots became white. Subjects' balance ability while standing on one or two legs improved during training sessions, whereas postural sway with open or closed eyes improved after the end of the study [64]. Moreover, participants declared that it was an enjoyable experience [64]. This claim can be a contributing factor in increasing treatment adherence. Even though balance training using video games and visual biofeedback has been shown to have promising effects on the postural balance of individuals with ASD, the lack of control groups limits the generalizability of the findings. So, future randomized controlled trials are needed.

### 3.6. Vestibular Therapy

Sensory integration therapy is a potential treatment designed to improve the processing of sensory inputs and their integration by providing appropriate sensory stimulations, especially proprioceptive, vestibular, and tactile stimuli [65–67]. A single 10-minute session of vestibular training using vestibular wings, a kind of sensory integration therapy, has positive effects on the postural sway of the people with ASD. A notable reduction of postural sway has also been shown during standing with eyes open on a plate, where the individuals utilize all sensory inputs to obtain postural balance [65]. However, the lack of a control group of children with ASD, inability to evaluate short-term effects, and the small sample size limits the generalizability of the findings. Hence, more research must be carried out to assess whether vestibular therapies are effective for individuals with ASD.

### 3.7. Transcranial Direct Current Stimulation

A deficit in sensory integration and its processing plays a role in the subsequent motor functions and balance control issues in those with ASD [68–70]. Transcranial direct current stimulation (tDCS) has neuromodulatory effects, changes the excitability of the neurons, and increases the activity of the brain cortex [71–74], which can enhance postural balance by improving sensory integration and subsequent improvement in motor function [69, 75]. Moreover, directing tDCS toward the motor cortex can enhance motor function by itself and, as a result, boost the effects of motor training [69, 76, 77]. In combination with motor training, these positive effects of tDCS have been utilized in 6–14-years-old children with ASD to improve their static and dynamic balance [69].

### 3.8. Sports, Play, and Active Recreation for Kids (SPARK)

SPARK is defined as a program to motivate children to engage in physical activity. Its lessons are associated with both health fitness and skill-fitness activities.

Regarding the previous knowledge about SPARK, Najafabadi et al. measured the effect of the SPARK program on motor impairment of ASD cases in the age range of 5–12 years. They found that twelve weeks of the SPARK program, three sessions per week, enhanced both social interactions and motor skills, including dynamic and static balance control and bilateral coordination, in 5–12-years-old children with ASD [78]. However, due to their small sample size and inability to blind participants to treatment allocation, more studies are required to decide whether SPARK is an effective treatment for poor balance control in ASD.

### 3.9. Square-Stepping Exercise (SSE)

SSE, including diverse movement patterns, has been organized to support lower extremity functional fitness. It has also been considered a fall-prevention exercise due to its effect on the agonist and antagonist muscles of the lower limbs [79, 80]. Barrios-Fernández et al. designed a study protocol to assess the effect of SSE on balance performance in individuals with ASD [79]. So, a randomized controlled trial is recommended to address whether SSE is a potential intervention for balance control in ASD.

## 4. Discussion

In this article, we provided a comprehensive summary of the available interventions to improve postural balance in individuals with ASD, which can be utilized by physical therapists, physicians, and researchers.

The current review has some practical implications for researchers, which can guide future studies on interventions improving postural balance in individuals with ASD. Although various types of interventions have been utilized to improve postural balance in individuals with ASD, studies evaluating the efficacy of these interventions to improve balance share some limitations. First, these studies designed interventions appropriate for children with ASD and mostly included children who were 14 years old or younger. ASD is a chronic condition that affects individuals throughout their lives [31, 32]. Balance impairment is also a problem, not only in children but also in adults with ASD [32]. Therefore, future studies should focus on evaluating the efficacy of interventions in older adults with ASD, and their needs should be considered in designing appropriate interventions to address their specific needs. The second limitation of the studies is their sample size, as most studies have small sample sizes. Such an issue reduced the generalizability of findings and exposed the studies to bias. Therefore, future studies with larger sample sizes are needed for a more precise assessment of the efficacy of the interventions. Third, the lack of control groups in some studies was another common limitation that should be addressed in future studies. Fourth, the long-term effects of the interventions should also be assessed in future studies, as they are not determined in current studies.

The present review has some clinical implications for the clinical context. Various systems have roles in maintaining postural balance [81]. Interventions designed to improve postural balance in individuals with ASD exert their effects utilizing different mechanisms (table 1). Therefore, these interventions can be utilized to address the individuals' specific needs. Also, considering the variation in the design of the interventions, from aquatic therapy to animal-assisted therapy, facilities may benefit from the interventions, which are affordable and feasible according to their available resources.

## 5. Conclusion

In this review, we sought to elucidate an up-to-date overview of the efficacy of the available interventions for motor impairment in ASD. To date, numerous studies have been conducted regarding the effect of a motor deficit on developing socio-communicative skills. However, an insufficient sample size and the lack of a control group in some studies can potentially compromise the validity of the results. Most interventions have been reported to affect the motor function of ASD through enhancing cerebellum or equilibrium function, sensory input integration and processing, muscle strength, and correcting body alignment. Due to the enjoyable features of some interventions, such as water-based exercises or video games for those with ASD, improved adherence to the treatment can be detected. The present review reached the preliminary conclusion that training programs designed for individuals with ASD could have some beneficial effects on their postural balance. Future well-designed randomized controlled trials are demanded to ascertain the possible effects of various interventions on balance performance in individuals with ASD.

## Figures and Tables

**Table 1:** Summary of studies evaluating the effects of physical interventions on balance performance in ASD.

Study	Participants	Intervention	Frequency	Results
Ansari et al., 2021 [23]	8–14-years-old boys with ASD at levels 1 or 2 of severity according to Gars-2	Training kata techniques and aquatic exercise	Ten weeks, two sessions per week, each session lasted for 60 minutes.	Static and dynamic balance improved in both intervention groups compared to controls.
Sarabzadeh et al., 2019 [24]	6–12-years-old children with ASD who had at least received one year of treatment for ASD	Tai Chi Chuan training	Ten weeks, two sessions per week, each session lasted for 60 minutes.	Static and dynamic balance improved in the intervention group compared to controls.
Kim et al., 2016 [29]	8–14-years-old children with ASD	Taekwondo	Eight weeks, two sessions per week, each session lasted for 50 minutes.	Performance in the right single-leg stance test with closed eyes improved compared to controls. There were no other significant differences in functional balance and postural sway during single- or double-leg stance tests with eyes open or closed between groups.
Yilmaz et al., 2004 [35]	A nine-years-old child with ASD	Swimming	Ten weeks	Performances of a patient with ASD in a single-leg stance test with eyes open or closed were improved.
Satiansukpong et al., 2008 [43]	Boys with ASD who were 11–19 years old	Elephant-assisted therapy	Three weeks, four days a week, each session lasted for seven hours.	Static and dynamic balance improved in four individuals with ASD.
Nuntanee et al., 2019 [42]	8–19-years-old children with ASD who had poor balance control	Motorizes elephant-assisted therapy	Four weeks, two days a week, each session lasted for 90 minutes.	Postural sway decreased in all directions in individuals who were in the experimental group; however, these changes were not statistically significant in all directions.
Ajzenman et al., 2013 [45]	5–12-years-old children with ASD	Hippotherapy	Twelve weeks, once a week, each session lasted for 45 minutes.	Postural control improved in individuals with ASD.
Wuang et al., 2010 [44]	6–10-years-old children with ASD	Simulated developmental horse-riding program	Twenty weeks, two sessions per week, each lasting for one hour.	Balance control significantly improved after combining occupational therapy and the simulated developmental horse-riding program compared to routine occupational therapy.
Lourenco et al., 2015 [57]	4–11-years-old children with mild to moderate ASD	Exercise on trampoline	Twenty weeks, one session per week, each session lasted for 45 minutes.	Functional dynamic balance improved more in individuals with ASD who exercised on a trampoline compared to controls.
Cheldavi et al., 2014 [60]	7–10-years-old boys with ASD and IQ of higher than 80	Progressive balance training	Six weeks, three sessions per week, each session lasting 45 minutes.	The velocity of the center of pressure and postural sway in anteroposterior and mediolateral directions decreased in individuals who participated in the program compared to controls.
Caldani et al., 2020 [62]	Children with ASD and a mean age of 11.7 years	Balance training using rehabilitation programs	Two sessions of dynamic balance training lasting for 45 minutes	Postural balance after a short postural rehabilitation training program was improved.
Travers et al., 2018 [64]	7–17-years-old individuals with ASD	Balance training using videogames	Six weeks, three sessions per week, each session lasted for an hour.	Postural sway with eyes open or closed and balance performances while standing on one or two legs were improved in participants.
Smoot Reinert et al., 2015 [65]	9–10-years-old children with ASD	Vestibular therapy	A session of ten minutes of vestibular therapy	Postural sway was decreased while standing on a plate with eyes open. Also, postural sway while standing on a foam pad with eyes closed was increased.
Mahmoodifar et al., 2020 [69]	6–14-years-old children with ASD and IQ of higher than 75	Transcranial direct current stimulation + motor training	Ten sessions of treatment	The dynamic and static balances were more improved in individuals who received transcranial direct current stimulation and motor training compared to those who only received exercise treatment
Najafabadi et al., 2018 [78]	5–12-years-old children with ASD	Sports, play, and active recreation for kids (SPARK)	Twelve weeks, three sessions per week	Both social interactions and motor skills, including dynamic and static balance control and bilateral coordination, have been enhanced.

## Data Availability

No data were used to support this study.
